# Global trends in Alzheimer’s disease and other dementias: A comprehensive analysis of incidence, socio-demographic variations, and future projections

**DOI:** 10.1371/journal.pone.0338018

**Published:** 2025-12-01

**Authors:** Ruixue Qin, Huijuan Zhao, Hui Gao, He Liu

**Affiliations:** 1 Department of Systems Science, Faculty of Arts and Sciences, Beijing Normal University, Zhuhai, Guangdong, China; 2 International Academic Center of Complex Systems, Beijing Normal University, Zhuhai, Guangdong, China; 3 School of Systems Science, Beijing Normal University, Beijing, China; 4 Department of Clinical Nutrition, Tongji Hospital, Tongji Medical College, Huazhong University of Science and Technology, Wuhan, Hubei, China; Kennesaw State University, UNITED STATES OF AMERICA

## Abstract

**Background:**

Alzheimer’s disease and other dementias (ADRD) are significant global health concerns, with rising incidence rates and substantial social and economic implications due to population aging.

**Methods:**

We investigated trends in ADRD incidence from 1992 to 2021 across age, sex, and socio-demographic index (SDI) regions, utilizing data from the Global Burden of Disease (GBD) 2021 platform. An age-period-cohort (APC) model was employed to analyze the effects of age, period, and birth cohort on ADRD incidence, and a Bayesian age-period-cohort (BAPC) model was used to predict future trends.

**Results:**

Globally, the age-standardized incidence rates (ASIR) remained relatively steady. However, the total number of ADRD cases witnessed a remarkable 141.25% increase, with 9,837,056 cases (95% UI: 8,620,519–11,163,700) in 2021. High SDI regions exhibited higher ASIR, whereas high-middle SDI regions showed the greatest growth, particularly among females. The net drift of ADRD incidence ranged from 0.43% per year in China to −0.68% per year in Denmark. Age effect was consistent across SDI regions, increasing exponentially with age. The 60–64 age group experienced the fastest annual incidence growth. High-middle SDI regions faced unfavorable period and cohort effects.

**Conclusion:**

Although progress in ADRD globally, significant regional and sex disparities persist. Strengthened surveillance and management of adults over 60 are urgently needed. Targeted public health policies and interventions are essential to address the escalating global dementia burden.

## Introduction

Alzheimer’s disease and other dementias (ADRD) are progressive neurodegenerative diseases, typically characterized by memory impairment and cognitive decline [1]. While the progression of ADRD varies, individuals typically survive 3.4 to 8.3 years after diagnosis, though some may live up to 20 years [2]. As of now, approximately 56.9 million people globally are living with ADRD, with projections suggesting that this number will soar to 152.8 million by 2050 [3]. ADRD’s cause-specific mortality ranking has risen from 18th in 1990–8th in 2021, underscoring the growing impact of these diseases on global health [4].

Age is the primary risk factor for ADRD, with prevalence rising sharply with age [5]. In the United States, about 10.8% of individuals aged 65 and older are affected by ADRD, and this figure increases with age: 5% for those aged 65–74, 13.1% for those aged 75–84, and 33.3% for those 85 and older [6]. Similar trends are seen in Europe, where the proportion of individuals with ADRD rises from 1.3% in those aged 65–69 to 40.8% in those aged 90 and older [7]. The global burden of young-onset dementia is also concerning, with a prevalence of 119 per 100,000 individuals, an incidence of 11 per 100,000, and around 3.9 million people aged 30–64 living with dementia, signaling a trend toward earlier onset [8].

Sex disparities in ADRD risk are pronounced, with women generally at higher risk, particularly those over 80 [9]. Some studies from the United States, European and Asian populations demonstrate a higher incidence of Alzheimer’s disease in women, while British studies suggest a higher incidence in men [10]. This sex disparity is likely due to a combination of factors, including biological differences, hormonal changes, and social determinants [11]. Women tend to experience faster disease progression, possibly due to longer life expectancy, declining estrogen levels and greater neurobiologic vulnerability [12]. However, these sex differences vary by age and geographic region.

Incidence refers to the number of new cases of a disease within a specific population over a defined period, and it is a critical measure of disease risk [13]. Some high-income countries have seen a decline in ADRD incidence over the past three decades [14], likely due to improvements in education [15] and the management of cardiovascular risk factors [16]. However, with the aging global population, increasing life expectancy, and the rising prevalence of conditions like diabetes and obesity [17], the burden of ADRD is expected to increase [18]. Particularly troubling is the rise in diabetes and obesity among younger populations, which could drive a rebound in ADRD incidence in the future [19].

Although no radical cure for ADRD currently exists, early diagnosis and timely interventions can help slow disease progression and improve quality of life [20]. Understanding trends in ADRD incidence is vital for forecasting the future burden of the disease and optimizing healthcare resource allocation. By examining how incidence varies across age, sex, and geographic regions, this study provides valuable insights into the evolving global health challenge posed by ADRD. We aim to estimate ADRD incidence trends from 1990 to 2021, using case numbers and age-standardized rates (ASR). We also employ an age-period-cohort (APC) model to assess temporal patterns and a Bayesian age-period-cohort (BAPC) model to predict future epidemiological trajectories.

## Methods

### Data sources

In Global Burden of Disease (GBD) 2021, Alzheimer’s disease and other dementias (ADRD) were defined using DSM and ICD criteria (ICD-10: F00–F03, G30–G31; ICD-9: 290, 294, 331). All ADRD data used in this analysis are publicly available through the GBD 2021 platform (https://ghdx.healthdata.org/gbd-2021/sources), which includes detailed information on data sources, statistical modeling, and methodologies as outlined in previous reports [21–22]. Incidence was estimated using the DisMod-MR 2.1 Bayesian meta-regression model. Detailed model specifications and covariates are provided in the supplementary method. For the first time, incidence data were included alongside prevalence and excess mortality. U.S. Marketscan claims data were excluded due to inconsistent case definitions and data volume imbalance. We analyzed global and regional trends in ADRD incidence from 1990 to 2021, with subgroup assessments by sex and age. Age-standardized incidence rates (ASIRs) were calculated to account for age-related confounding and ensure comparability across populations. Given the limited number of ADRD cases in individuals under 40 years, the study primarily focused on individuals aged 40 and above.

The Socio-demographic Index (SDI) used in this analysis is a composite measure that incorporates three components: per capita income, average years of schooling, and fertility rates among females aged under 25 years. SDI values range from 0 to 1, with higher values indicating greater socioeconomic development. Based on GBD 2021 data, countries and territories were classified into five SDI quintiles: low, low-middle, middle, high-middle, and high SDI regions.

### Analysis of overall trends in ADRD incidence

We examined trends in ADRD incidence, focusing on both the total number of cases and the corresponding age-standardized incidence rate (ASIR) with 95% uncertainty intervals (UIs) from 1990 to 2021. The data were classified by sex, age, year, SDI, and 204 countries and territories. ASIRs were calculated using global age-standardized population data from GBD 2021. We also analyzed the age distribution of incidence cases, grouping participants into 12 age brackets ranging from 40–44 years to >95 years. To reflect disease fluctuations across different age groups, the proportion of incidence cases in each age group was calculated.

To analyze temporal patterns of ASIR from 1992 to 2021, we estimated the annual percentage change (EAPC) with 95% confidence intervals (CIs). By fitting the natural logarithm of the age-standardized rate to the calendar year, the EAPC is calculated to describe the long-term trend of the disease burden ASR. An increasing trend in ASIR was indicated when both the EAPC and its lower confidence limit exceeded 0. Conversely, a declining trend was signified when both the EAPC and its upper confidence limit fell below 0.

### Age-period-cohort (APC) model analysis of incidence data

The Age-Period-Cohort (APC) model is a statistical tool used in epidemiology, demography, and social sciences to disentangle and analyze the influence of three distinct temporal factors on a specific outcome or trend: age effects, period effects, and cohort effects [23]. The age effect refers to the risk associated with different age groups, the period effect reflects how changes over time impact all age groups, and the cohort effect examines how outcomes evolve over time for individuals born in the same period.

We applied the APC model to analyze the changing trends of ADRD incidence across different age groups, time periods, and birth cohorts, disaggregated by sex and SDI regions, using data from 1992 to 2021. Age intervals were congruent with period intervals, with 5-year age groups paired with corresponding 5-year periods. Specifically, age was stratified into 12 age groups (from 40–44 years to >95 years), and time was divided into six 5-year periods: 1992–1996, 1997–2001, 2002–2006, 2007–2011, 2012–2016, and 2017–2021. As a result, the analysis covered 17 overlapping birth cohorts, with birth years spanning from 1897 to 1977.

The APC model estimated longitudinal age-specific incidence rates, period effects, and cohort effects. The results included the overall annual percentage change (net drift, % per year) and age-specific annual percentage change (local drift, % per year). The significance of these trends was tested using the Wald chi-square test. The age effect was represented by the longitudinal age-specific incidence rates, while the period and cohort effects were expressed as relative risks by comparing age-specific incidence rates for each period/cohort with those of the reference period/cohort. The selection of the reference period/cohort was arbitrary and did not impact the interpretation of the results. All statistical tests were two-sided, with a p-value of <0.05 considered significant. Data analyses were conducted using R statistical software (version 4.4.1).

### Bayesian age-period-cohort (BAPC) model

The future incidence of ADRD from 2022 to 2036 was predicted using the Bayesian Age-Period-Cohort (BAPC) model with integrated nested Laplace approximations (INLA) [24]. The BAPC model generates age-standardized incidence rates and allows for the prediction of ADRD trends, considering the effects of age, period, and cohort. We utilized data from the year 2021, acknowledging that the statistics for this period may have been directly impacted by the COVID-19 pandemic. However, it is important to emphasize that the effects of COVID exhibit long-term persistence, and its associated health burden is likely to endure within disease prevalence trends.

## Results

### Global and SDI regional trends in ADRD incidence (1992–2021)

Table 1 summarizes the incidence numbers, age-standardized incidence rates (ASIR), estimated annual percentage change (EAPC) of ASIR, and net drift of ADRD incidence. Over the past 30 years, the global incidence of ADRD has more than doubled, increasing from 4,077,542 cases (95% UI: 3,587,065–4,630,267) in 1992–9,837,056 cases (95% UI: 8,620,519–11,163,700) in 2021—a 141.25% rise. However, the global ASIR for ADRD in 2021 (119.76 per 100,000 population; 95% UI: 104.96–135.89) remains comparable to that in 1992 (117.69; 95% UI: 103.69–133.01), reflecting an EAPC of −0.03 (95% UI: −0.06 to −0.01) and a net drift of 0.01 (95% CI: 0.00–0.02). These findings suggest that while the absolute number of cases has risen significantly, the overall ASIR has remained relatively stable, indicating a balance between increased incidence and population growth.

**Table 1:** Trends in ADRD incidence across socio-demographic Index quintiles,1992−2019.

		1992		2021		1992 - 2021		
	Sex	Incidence Number (N, 95% UI)	Incidence ASR (per 100 000, 95% UI)	Incidence Number (N, 95% UI)	Incidence ASR (per 100 000, 95% UI)	Percent change of number (%)	EAPC of ASIR (%, 95% CI)	Net Drift (%/year)
**Global**	Both	4077542 (3587065,4630267)	117.69 (103.69,133.01)	9837056 (8620519,11163700)	119.76 (104.96,135.89)	141.25	−0.03 (−0.06 to −0.01)	0.01 (0.00 to 0.03)
	Female	2639001 (2322742,2997830)	128.85 (113.83,145.2)	6191564 (5432752,7009226)	132.29 (116.3,149.8)	134.62	−0.01 (−0.04 to 0.01)	0.02 (0.01 to 0.04)
	Male	1438541 (1252543,1645622)	101.07 (88.5,114.99)	3645492 (3144738,4183541)	103.4 (89.45,118.45)	153.42	0.01 (−0.01 to 0.03)	0.04 (0.01 to 0.06)
**Socio-Demographic Index (SDI)**								
Low SDI	Both	151469 (132165,171831)	94.64 (82.4,107.72)	328703 (287112,372981)	90.89 (79,103.12)	117.01	−0.18 (−0.2 to −0.17)	−0.19 (−0.21 to −0.16)
	Female	88096 (76930,99818)	108.76 (94.88,123.38)	196356 (171792,222478)	103.42 (90,117.04)	122.89	−0.21 (−0.21 to −0.2)	−0.21 (−0.24 to −0.18)
	Male	63374 (55039,72097)	79.85 (69.38,91.32)	132347 (115523,150718)	76.65 (66.62,87.44)	108.83	−0.21 (−0.23 to −0.19)	−0.21 (−0.24 to −0.17)
Low-middle SDI	Both	439242 (384345,498412)	95.21 (83.01,108.34)	1063277 (929612,1207790)	92.61 (80.79,105.71)	142.07	−0.17 (−0.19 to −0.15)	−0.13 (−0.15 to −0.12)
	Female	246332 (215733,279154)	105.46 (92.07,119.68)	623194 (546496,706851)	100.27 (87.57,114.21)	152.99	−0.23 (−0.25 to −0.21)	−0.20 (−0.21 to −0.18)
	Male	192910 (168603,219647)	84.51 (73.46,96.51)	440083 (384919,501587)	83.35 (72.46,95.38)	128.13	−0.14 (−0.17 to −0.12)	−0.10 (−0.12 to −0.08)
Middle SDI	Both	926754 (809182,1056770)	115.73 (101.29,131.64)	2902080 (2542320,3314767)	123.79 (108.25,141.26)	213.14	0.07 (0.02 to 0.11)	0.06 (0.03 to 0.08)
	Female	568914 (497782,646980)	129.11 (113.52,146.64)	1795519 (1574345,2041903)	137.75 (120.93,156.84)	215.60	0.04 (−0.01 to 0.09)	0.03 (0.00 to 0.06)
	Male	357840 (309953,408695)	98.46 (85.77,112.71)	1106561 (949936,1274802)	106.23 (91.67,122.15)	209.23	0.13 (0.09 to 0.16)	0.09 (0.07 to 0.12)
High-middle SDI	Both	1054854 (919298,1211423)	119.71 (104.93,135.89)	2582346 (2252029,2941774)	132.4 (115.43,150.85)	144.81	0.21 (0.17 to 0.25)	0.21 (0.18 to 0.24)
	Female	709479 (616685,812076)	128.81 (113.1,145.74)	1678920 (1469521,1904993)	145.37 (127.62,165.13)	136.64	0.27 (0.22 to 0.32)	0.25 (0.22 to 0.28)
	Male	345375 (297130,397662)	104.17 (90.54,119.22)	903427 (771153,1042120)	114.05 (97.8,131.27)	161.58	0.20 (0.17 to 0.24)	0.20 (0.16 to 0.25)
High SDI	Both	1500526 (1326297,1690270)	127.05 (112.62,142.25)	2952147 (2586722,3344548)	122.61 (107.45,138.44)	96.74	−0.11 (−0.12 to −0.1)	0.00 (−0.04 to 0.03)
	Female	1023164 (907109,1152775)	136.48 (121.71,152.57)	1892156 (1676948,2140304)	135.14 (119.13,152.48)	84.93	−0.03 (−0.05 to −0.01)	0.06 (0.02 to 0.11)
	Male	477363 (415509,543812)	111.26 (97.8,125.8)	1059991 (914025,1211000)	106.34 (92.24,121.37)	122.05	−0.12 (−0.14 to −0.1)	−0.02 (−0.06 to 0.01)

**Fig 1:**
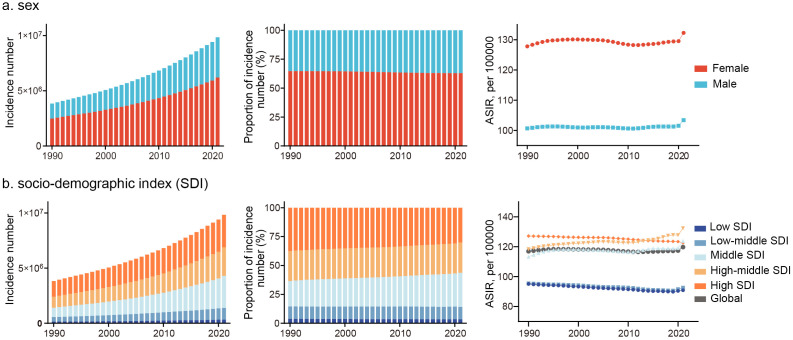
Temporal change in the numbers, proportion and ASIR of ADRD incidence across (a) sex and (b) SDI quintiles from 1990 to 2021.

Females consistently exhibit higher ASIR than males and this pattern persisted across all SDI regions (Fig 1A and S1 Fig in S1 File). In high-middle SDI regions, both the EAPC (females: 0.27, males: 0.20) and net drift (females: 0.25, males: 0.20) indicated a more pronounced increase among females. In contrast, low and middle SDI regions demonstrated a narrowing sex gap (Table 1). These findings underscore the sex disparities in ADRD incidence, which may have important implications for healthcare planning and resource allocation.

Across the five SDI regions, ADRD incidence has steadily increased since 1992. Fig 1B shows how incidence has shifted across these regions over time, with high and high-middle SDI countries consistently accounting for more than half of the global cases. Over this period, the proportion of cases in middle SDI regions has gradually increased, signaling a shifting burden of disease. Notably, ASIR spiked in 2021, likely due to the impact of the COVID-19 pandemic. This suggests that pandemic-related factors may have influenced ADRD diagnoses, warranting further investigation into COVID-19’s long-term effects on neurodegenerative disease incidence.

ASIR trends have consistently exceeded the global average in high-middle and high SDI regions, while trends in middle SDI regions closely mirror global patterns. Conversely, low and low-middle SDI regions have seen the lowest ASIR values. Net drift analysis reveals significant declines in ADRD incidence in low (−0.19; 95% CI: −0.21 to −0.16) and low-middle (−0.13; 95% CI: −0.15 to −0.12) SDI regions. On the other hand, incidence rates have increased in high-middle (0.21%; 95% CI: 0.18–0.24) and middle (0.03%; 95% CI: 0.03–0.08) SDI regions. Notably, the EAPC and net drift results do not align in high SDI regions. While standardized incidence rates have declined, overall population changes remain small, indicating variations in trends across different age groups.

### National trends in ADRD incidence (1992–2021)

**Fig 2:**
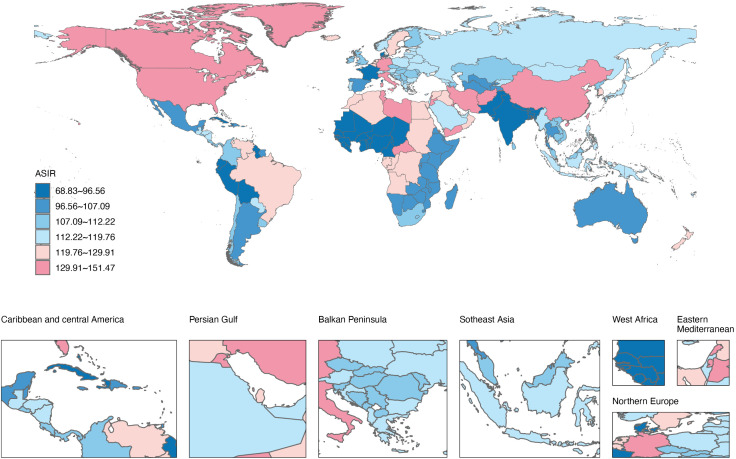
World map of age-standardized incidence rate in 2021 for ADRD in 204 countries and territories. Reprinted from *Resource and Environmental Science Data Platform* [https://www.resdc.cn], under a CC BY license, with permission from the *Institute of Geographic Sciences and Natural Resources Research, Chinese Academy of Sciences*, original copyright © 2014-2025.

**Fig 3:**
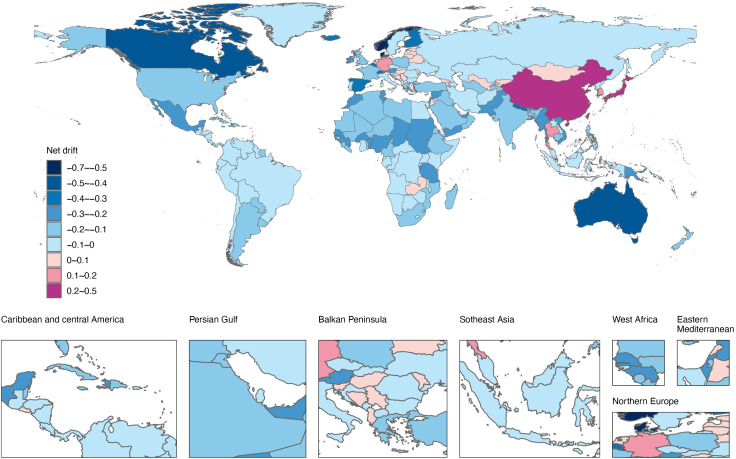
World map of net drift of incidence during 1992 − 2021 for ADRD in 204 countries and territories. Reprinted from *Resource and Environmental Science Data Platform* [https://www.resdc.cn], under a CC BY license, with permission from the *Institute of Geographic Sciences and Natural Resources Research, Chinese Academy of Sciences*, original copyright © 2014-2025.

Figs 2−3, S2 Fig and S1 Table in S1 File present the incidence numbers, ASIR, EAPC, and net drift for 204 countries and territories from 1992 to 2021. In 2021, 17 countries reported more than 100,000 cases of ADRD, cumulatively accounting for 75.65% of global incidence. Notably, China, the United States, and India ranked as the top three countries in terms of ADRD incidence cases.

Forty-two countries had ASIRs higher than the global average (119.76 cases per 100,000), with China, Germany, Lebanon, Turkey, and Greenland among the highest. Conversely, the countries with the lowest ASIRs included Nigeria, Sao Tome and Principe, and Ghana. Net drift analysis revealed that 33 countries exhibited an increasing trend in ADRD incidence (net drift > 0.0%), with China (0.43%) and Taiwan (0.40%) showing the highest increases. Denmark, however, experienced the greatest decrease (−0.68%). In most countries and territories, the ASIRs remained relatively stable and even showed downward trends (net drift < 0.0%).

Overall, the analysis of the correlation between ASIR and the SDI from 1992 to 2021 revealed positive trend (ρ = 0.38, p < 0.001) across GBD 21 regions. The ASIRs of 204 countries were also positively correlated with SDI levels in 2021 (ρ = 0.34, *p* < 0.001), highlighting the role of socio-economic development in shaping ADRD incidence patterns. Cluster analysis of net drift and SDI across 204 countries and territories demonstrated significant heterogeneity in ASIR changes among higher SDI regions: notable upward trends were observed in China and Japan, while Denmark and Norway experienced substantial declines. Moderate ASIR decreases characterized middle- and low-SDI regions overall (S4 Fig in S1 File).

### Time trends in ADRD incidence across different age groups

**Fig 4:**
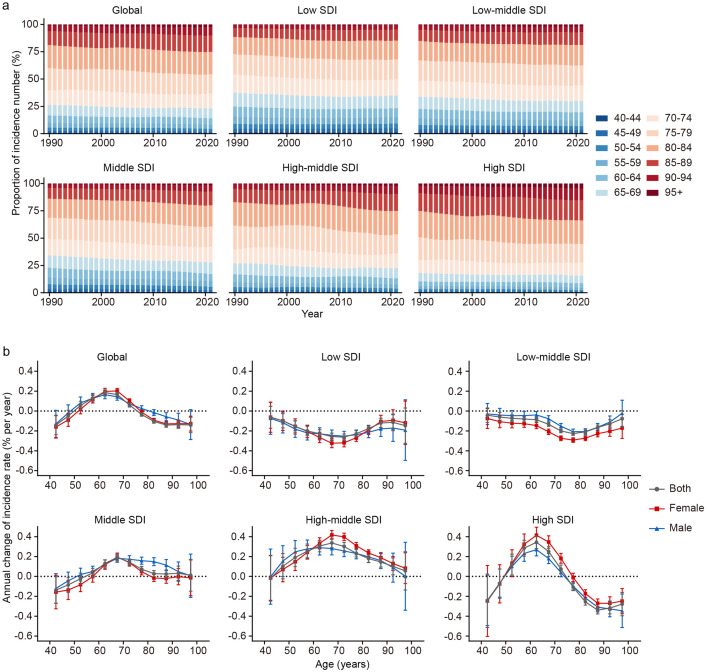
Age distribution and local drifts of ADRD incidence by SDI quintiles. **(a)** Temporal change in age distribution of ADRD incidence numbers from 1990 to 2021. **(b)** Local drifts of ADRD incidence (estimates from age-period-cohort models) for 12 age groups from 1992 to 2021. The dots and error bar indicate the annual percentage change (% per year) and the corresponding 95% CIs.

Fig 4A shows the age distribution trends of ADRD incidence over time in different SDI regions. The incidence numbers in all age groups have shown an upward trend, but the proportion of each age group varies by region. Globally, ADRD incidence has gradually shifted from younger to older populations, a trend more pronounced in middle, high-middle, and high SDI regions. Notably, individuals over 70 accounted for the largest proportion of cases in higher SDI regions, while lower SDI regions exhibited a younger age distribution. The age distribution patterns for males and females across SDI quintiles are presented in S3 Fig in S1 File.

Fig 4B highlights local drifts in ADRD incidence across age groups, calculated through the APC model. Globally, the incidence increased in the 50–54–70–74 age groups, with the most significant rise observed in the 60–64 age group (local drift: 0.18; 95% CI: 0.16–0.21). In contrast, the remaining age groups exhibited a general decline.

Age-specific trends varied across SDI regions. In high SDI regions, incidence increased in the 50–54–70–74 age groups, while a decline was observed in those over 80. The high-middle SDI region exhibited rising incidence in age groups above 45, with the most notable increase in the 65–69 age group (0.34; 95% CI: 0.29–0.38). Conversely, low and low-middle SDI regions showed declining incidence across all age groups, further emphasizing the disparities between higher and lower SDI regions.

### Age, period, and cohort effects on ADRD incidence

**Fig 5:**
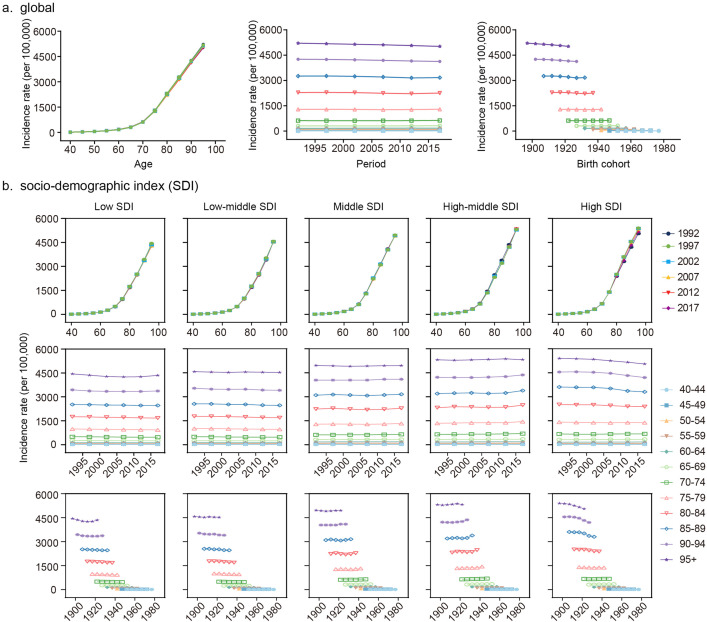
Long-term trends of age-specific, period-based, and cohort-based variation of ADRD incidence across (a) global and (b) SDI quintiles during 1992-2021.

**Fig 6:**
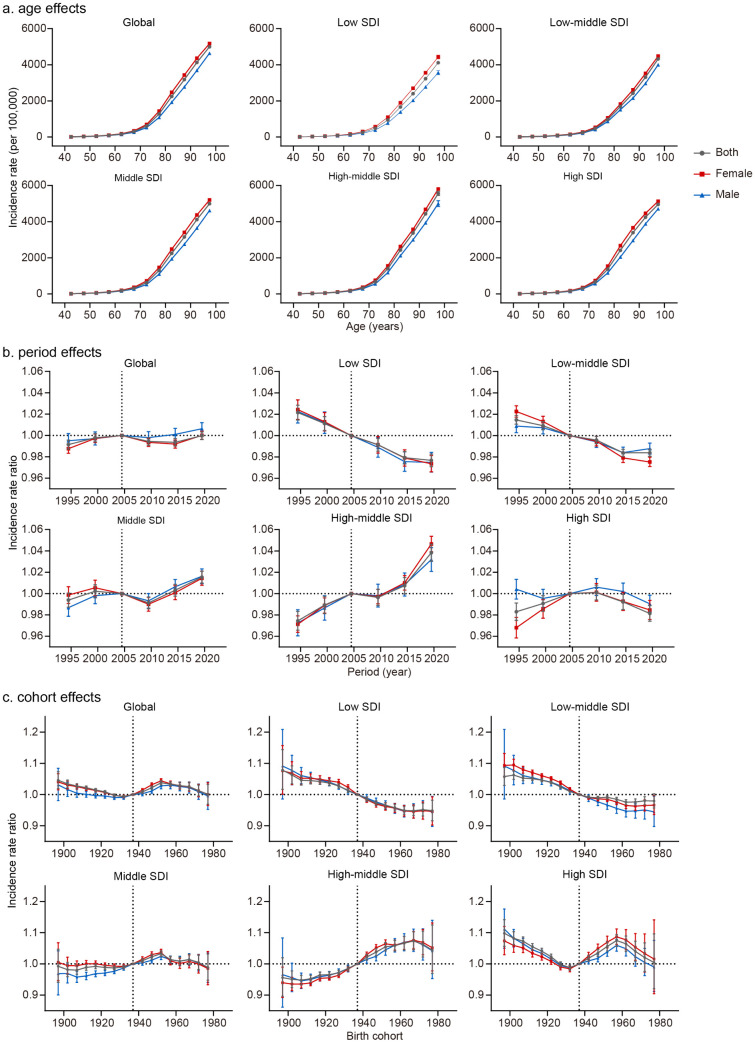
Age, period and cohort effects on ADRD incidence by SDI quintiles. **(a)** Age effects are shown by the fitted longitudinal age curves of incidence rate (per 100,000) adjusted for period deviations. **(b)** Period effects are shown by the relative incidence rate and computed as the ratio of age-specific rates from 1992-1996 to 2017 2021, with the reference period from 2002 to 2006. **(c)** Cohort effects are shown by the relative risk of incidence rate and computed as the ratio of age-specific rates from the 1897 cohort to the 1977 cohort, with the referent cohort set at 1937. The dots and error bar indicate incidence rates or rate ratios and the corresponding 95% CIs.

Figs 5−6 and S2 Table in S1 File present the estimated age-period-cohort (APC) effects on ADRD incidence across different SDI regions. Age effects were consistent across regions, with incidence risk increasing with age and rising sharply after 70. Low and low-middle SDI regions had lower incidence rates across all age groups. Interestingly, males exhibited smaller age-related increases in disease risk than females, indicating sex differences in disease susceptibility. The analysis of age-specific incidence trends describes in detail the change characteristics of each age group across different time periods and birth cohorts, and the trend manifestation is consistent with the results of local drift (Fig 5).

Period effects remained largely unchanged globally over the past three decades, indicating limited improvement in prevention strategies. Period effects declined in low and low-middle SDI regions but increased significantly in high-middle SDI regions, suggesting emerging challenges as economies develop. Cohort effects varied by SDI region. In high SDI regions, cohort risk remained stable with slight fluctuations. However, in high-middle SDI regions, risk increased for younger birth cohorts, whereas in low and low-middle SDI regions, cohort risk decreased. In middle SDI regions, risk rose for cohorts born before 1952 but declined for those born afterward.

Both sexes exhibited distinct temporal patterns in ADRD incidence across SDI regions. Overall, sex disparities were most pronounced in high SDI regions, where males showed greater susceptibility to period effects. In terms of cohort effects, males had higher rate ratio than females in older birth cohorts before reference cohort 1937, whereas females surpassed males in later cohorts. This finding further supports that discrepancies between EAPC and net drift analyses in high-SDI regions reflect age-specific trend heterogeneity, consistent with the patterns observed in local drift values across age groups.

### Predicted trends of ADRD incidence to 2036

**Fig 7:**
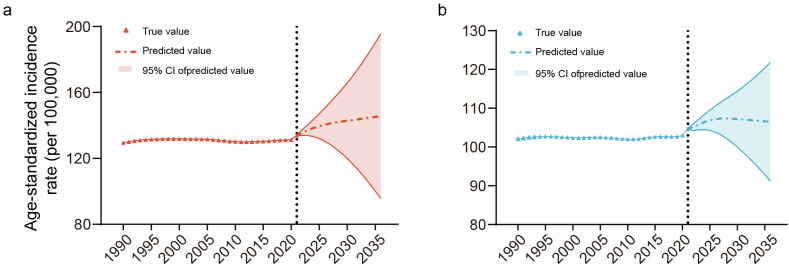
Predicted trends of ADRD incidence globally to 2036. The (a) red and (b) blue triangles indicate the true value of ASIR from 1990 to 2021 for female and male, the dotted lines and the light color regions indicate the predicted value and the corresponding 95% CIs.

Using the Bayesian Age-Period-Cohort (BAPC) model, we predicted ADRD incidence trends from 2022 to 2036 based on data from 1990 to 2021 (Fig 7). The model forecasts a continued upward trend in ASIR for both sexes, with females consistently exhibiting higher rates than males. Females’ ASIR is expected to rise from 134.04 per 100,000 in 2021 to 145.60 per 100,000 in 2036, while males’ ASIR will show a modest increase from 104.82 to 106.52 per 100,000. The BAPC model predicts a significant overall increase in ADRD incidence, particularly among females.

## Discussion

Over the past three decades, the ASIR of ADRD has remained relatively stable, while the total number of ADRD cases has risen significantly due to population growth. Notably, there are significant differences in ASIR across SDI regions, with high-SDI regions consistently showing higher incidence rates compared to low SDI regions. Age remains a key independent risk factor for AD, with incidence increasing exponentially as individuals age. In particular, high-middle SDI regions have exhibited unfavorable period and cohort effects, indicating an increasing future disease burden. Our predictive models suggest that the global ASIR will continue to rise through 2036, especially among women. These trends underscore the challenges of aging populations and regional variations, highlighting the urgent need for tailored healthcare policies and public health interventions to manage the growing global burden of dementia.

Our analysis revealed notable sex and regional patterns in ADRD incidence. The rapid increase among females in high-middle SDI regions likely reflects both demographic ageing and improved diagnostic capacity. The observed sex-specific temporal variations suggest that males in high SDI regions were more influenced by period effects, while females exhibited stronger cohort-related increases in younger generations. Meanwhile, the narrowing sex gap in lower SDI regions may reflect limited healthcare awareness and lower diagnostic detection. The observed sex and regional differences in ADRD incidence highlight the multifactorial nature of risk. Biologically, the longer life expectancy and hormonal changes after menopause of females may increase susceptibility to neurodegenerative processes [25]. Social and behavioral factors, including disparities in education, healthcare access, and caregiving patterns, may also contribute [26].

Among the 33 countries with positive net drift values, 11 were high SDI and 10 were high-middle SDI regions. The top five countries were China, Japan, South Korea, Germany, and Thailand, with a notable concentration of Asian countries. China ranks first in terms of ADRD cases, incidence, net drift value, and EAPC of ASIR. Between 1990 and 2021, the number of cases in China increased by 265.24%, accounting for 29.62% of the global total, while its incidence rate rose by 19.29%. This rapid increase is driven by China’s large population and its accelerating aging demographic, and the population of people over 60 is projected to reach 28% by 2040 [27]. In contrast, nine of the ten countries with the fastest-declining ASIR (net drift < 0) were high SDI regions, primarily European countries like Denmark, Norway, Luxembourg, and Finland. This trend likely reflects successful prevention strategies in these regions. For example, Denmark promotes international dementia research collaborations [28], while Norway ensures high-quality care through universal healthcare, substantial home care subsidies, and advanced geriatric facilities [29]. According to the WHO’s 2021 Global Status Report on the Public Health Response to Dementia, only 25% of countries have national dementia strategies, with half of these initiatives concentrated in Europe [30].

WHO has identified nine primary risk factors for dementia: aging, hypertension, hyperglycemia (diabetes), overweight/obesity, smoking, excessive alcohol consumption, physical inactivity, social isolation, and depression [31]. Our study shows a strong positive correlation between ASIR and SDI, with higher incidence rates in high SDI regions likely due to increased life expectancy and the accumulation of risk factors associated with economic development. The decline in ASIR in low SDI regions may be attributed to shorter life expectancies, underdiagnosis, and lifestyle differences rather than a true reduction in risk [32]. Economic disparities, policy priorities, and cultural differences across countries complicate interpretations, although no universal explanatory framework has been established.

Our study also employed the age-period-cohort (APC) model to examine the temporal dynamics of ADRD incidence. A consistent age effect was found across all SDI regions, with risk increasing significantly as individuals age, especially for populations over 65, where female incidence rates consistently exceed male rates. Analysis of local drift values revealed the fastest annual growth in the 60–64 age group, which could be linked to earlier disease onset or improvements in diagnostic capabilities. Globally, fluctuating period and cohort effects were observed. High SDI regions showed a downward trend in these effects during the latter part of the study period. This may be due to improved healthcare resource allocation, early detection of dementia risk factors, and increased educational investments in these regions [33]. Conversely, rising period and cohort effects in high-middle SDI regions may be associated with socioeconomic development and greater exposure to traditional risk factors [3]. These findings highlight the need for region-specific prevention and control strategies in high-middle SDI regions, including increased public awareness and optimized medical resource distribution to mitigate the growing disease burden.

Previous studies have primarily provided comprehensive analyses of the prevalence, incidence, mortality, and DALYs of ADRD. Many of these studies focused on specific aspects, such as age-specific trends in younger [8] or older populations [34], regional patterns in high-burden areas like Asia [35] and India [36], or the role of risk factors including metabolic risks [37] and lifestyle behaviors [38]. In this study, we focused on incidence to systematically assess temporal trends in ADRD using statistical models that can separate age, period and cohort effects. Our findings are broadly consistent with previous studies, confirming that ADRD burden increases with population aging and is higher among females. However, by incorporating SDI stratification and sex differences, our analysis provides a more detailed understanding of global patterns and inequalities in ADRD incidence, revealing long-term temporal shifts, generational vulnerabilities, and the influence of socioeconomic development on disease dynamics. This study extends previous findings by demonstrating how the temporal dynamics of ADRD differ by sex and socioeconomic level, highlighting an epidemiological shift of the dementia burden toward rapidly developing regions.

While this study provides critical insights into the trends of ADRD incidence, it has limitations. Incomplete ADRD surveillance systems in low- and middle-income countries could result in data gaps and underreporting. Heterogeneity in diagnostic criteria and undetected cases in resource-limited regions may introduce bias in incidence estimates. Moreover, reliance on indirect GBD modeling may cause measurement errors due to missing primary data, affecting the accuracy of age/period/cohort effect estimates. Additionally, the GBD classification system prevents the differentiation of dementia subtypes, limiting our understanding of the specific contributions of each subtype to overall disease burden. Future research should integrate complementary data sources, such as clinical studies, genetic research, and longitudinal datasets, to provide a more comprehensive understanding of ADRD epidemiology.

## Conclusion

Our study applied an age–period–cohort model to the GBD data on ADRD incidence, enabling a deeper understanding of the temporal dynamics of disease incidence across age groups, periods, and birth cohorts. While the global age-standardized incidence rate of ADRD remained stable, the number of cases increased by over 140%. The increasing burden, particularly in high-middle SDI regions and among females, reflects aging and socio-economic transitions. Unfavorable period and cohort effects were observed, with the fastest increase in incidence among individuals aged 60–64. Regional differences underscore the need for tailored public health strategies and targeted interventions, including early detection and age-specific prevention, especially in high-middle SDI regions and among ageing populations.

## Supporting information

S1 File Supporting information. This file contains all supplementary figures and tables, including Figure S1–S4 and Table S1–S2. Detailed descriptions of each figure and table are provided within the file.
(DOCX)
