# Supplementary material for: Global trends in Alzheimer’s disease and other dementias: A comprehensive analysis of incidence, socio-demographic variations, and future projections
Source: PLoS One. 2025 Dec 1;20(12):e0338018. doi: 10.1371/journal.pone.0338018 (PMC12668476; doi:10.1371/journal.pone.0338018)
Supplement: S1 File — This file contains all supplementary figures and tables, including Figure S1–S4 and Table S1–S2. Detailed descriptions of each figure and table are provided within the file. (DOCX) [file pone.0338018.s001.docx]

**Supplementary Method**

**DisMod-MR 2.1 Bayesian meta-regression model**

The epidemiological parameters for Alzheimer's disease and other dementias were derived using DisMod-MR 2.1 model, a Bayesian meta-regression tool developed for the Global Burden of Disease (GBD) study [1]. This model synthesizes disparate data on incidence, prevalence and mortality to produce internally consistent estimates across different populations, time periods and geographies.

For the GBD 2021 dementia analysis, two distinct DisMod-MR models were run. Model 1 incorporated prevalence, within-cause mortality, and relative risk data to establish the baseline epidemiological structure. Model 2 integrated adjusted prevalence, incidence, and cause-specific mortality after excluding dementia cases attributable to other causes (e.g., stroke, Parkinson’s disease, traumatic brain injury, Down syndrome, and HIV). The two-stage framework allowed for mortality adjustment and alignment with the overall dementia impairment envelope, producing the final GBD estimates for ADRD.

Covariates were incorporated to help the model extrapolate to data-sparse regions and account for heterogeneity between data sources. Age-standardized education and smoking prevalence (both sexes combined) were included as country-level covariates in the DisMod-MR 2.1 model, representing cognitive reserve and behavioral risk exposure, respectively, with evidence indicating that higher education is protective and smoking is positively associated with dementia risk.

[1] GBD 2019 Diseases and Injuries Collaborators. Global burden of 369 diseases and injuries in 204 countries and territories, 1990–2019: a systematic analysis for the Global Burden of Disease Study 2019. The Lancet 2020;396: 1204–22. doi:[10.1016/S0140-6736(20)30925-9](https://doi.org/10.1016/S0140-6736(20)30925-9)

**S1 Table.** Time trends in ADRD incidence for both sexes in 204 countries and territories, 1992-2019

|  | 1992 | | 2021 | | 1992-2021 | |
| --- | --- | --- | --- | --- | --- | --- |
| location | **Incidence Number  (N, 95% UI)** | **Incidence ASR  (per 100 000, 95%UI)** | **Incidence Number  (N, 95% UI)** | **Incidence ASR  (per 100 000, 95%UI)** | **EAPC of ASIR  (%, 95%CI)** | **Net drift  (%)** |
| China | 797869 (685680,915875) | 126.98 (110.73,144.66) | 2914112 (2504728,3350743) | 151.47 (131.22,173.34) | 0.36 (0.29,0.43) | 0.43 |
| Taiwan (Province of China) | 11457 (9903,13141) | 90.76 (78.7,104.03) | 43204 (36980,48565) | 98.1 (83.69,110.2) | 0.41 (0.31,0.51) | 0.40 |
| Japan | 200071 (174105,229289) | 115.42 (100.73,131.35) | 576270 (503457,662794) | 117.23 (102.05,133.68) | 0.2 (0.14,0.26) | 0.26 |
| Republic of Korea | 29055 (25309,32819) | 126.55 (111.98,143.2) | 117489 (102910,133127) | 124.63 (109.44,140.89) | 0 (-0.06,0.06) | 0.11 |
| Germany | 204241 (182945,226402) | 145.4 (130.74,160.44) | 339825 (299328,382749) | 142.13 (124.57,159.72) | -0.11 (-0.14,-0.07) | 0.10 |
| Thailand | 29905 (26103,33962) | 101.98 (88.82,115.8) | 112595 (98738,127875) | 103.27 (90.64,117.58) | 0.13 (0.07,0.19) | 0.10 |
| Mongolia | 948 (823,1083) | 114.31 (100.16,130.48) | 1819 (1599,2051) | 115.83 (101.21,132.52) | 0.06 (0.05,0.07) | 0.08 |
| Kyrgyzstan | 2955 (2576,3359) | 112.64 (98.22,128.38) | 4134 (3622,4676) | 113.88 (99.39,130.07) | 0.06 (0.05,0.07) | 0.07 |
| Netherlands | 27847 (24500,30791) | 127.03 (112.09,139.74) | 48272 (42168,54730) | 121.79 (106.84,137.86) | -0.14 (-0.17,-0.11) | 0.07 |
| Slovenia | 2750 (2387,3154) | 109.95 (95.52,125.95) | 5733 (4981,6582) | 110.51 (96.63,126.67) | 0.05 (0.02,0.08) | 0.07 |
| Latvia | 4060 (3525,4677) | 114.12 (99.75,130.94) | 5467 (4733,6277) | 114.97 (99.97,131) | 0.05 (0.02,0.07) | 0.06 |
| El Salvador | 3296 (2903,3732) | 110.78 (97.12,126.07) | 7619 (6700,8708) | 112.22 (98.14,128.45) | 0.07 (0.05,0.08) | 0.06 |
| Maldives | 68 (58,77) | 109.88 (95.92,125.48) | 309 (269,355) | 113.22 (98.24,129.67) | 0.11 (0.08,0.14) | 0.06 |
| Albania | 1929 (1688,2194) | 111.73 (97.26,127.96) | 4828 (4174,5529) | 112.62 (98,128.7) | 0.04 (0.03,0.05) | 0.06 |
| Nauru | 3 (3,4) | 111.9 (96.21,129.18) | 4 (4,5) | 113.97 (98.71,131.23) | 0.03 (0,0.07) | 0.05 |
| Republic of Moldova | 3958 (3414,4538) | 110.9 (96.49,126.27) | 6801 (5910,7736) | 111.97 (97.12,128.21) | 0.02 (0,0.04) | 0.05 |
| Jordan | 1298 (1131,1476) | 132.21 (114.69,151.07) | 7243 (6314,8274) | 134.51 (116.99,153.81) | 0.09 (0.06,0.13) | 0.05 |
| Rwanda | 1968 (1701,2235) | 106.36 (93,121.52) | 4609 (4024,5193) | 106.57 (93.24,120.76) | 0.05 (0.03,0.07) | 0.05 |
| Micronesia (Federated States of) | 43 (37,51) | 119.51 (103.39,137) | 56 (48,64) | 120.71 (104.84,139.46) | 0.07 (0.06,0.07) | 0.05 |
| Singapore | 1709 (1490,1914) | 91.19 (80.55,101.93) | 7764 (6988,8584) | 94.88 (85.54,104.85) | 0.16 (0.14,0.19) | 0.05 |
| Estonia | 2264 (1941,2617) | 113.71 (98.59,130.74) | 3662 (3185,4204) | 112.78 (98.89,128.28) | -0.02 (-0.07,0.03) | 0.04 |
| Serbia | 9626 (8222,11051) | 111.52 (97.2,127.83) | 19543 (16949,22543) | 111.65 (96.91,128.06) | -0.01 (-0.02,0) | 0.04 |
| Kiribati | 29 (25,33) | 121.44 (105.53,138.95) | 54 (46,62) | 121.84 (106.12,138.96) | -0.01 (-0.04,0.02) | 0.04 |
| Zambia | 2064 (1795,2346) | 101.93 (88.76,115.9) | 4723 (4112,5326) | 102.65 (89.99,117.22) | 0.07 (0.04,0.09) | 0.03 |
| Bosnia and Herzegovina | 3735 (3220,4293) | 111.74 (97.28,128.13) | 7140 (6148,8275) | 111.79 (96.79,127.27) | 0 (-0.03,0.04) | 0.03 |
| Georgia | 6594 (5771,7525) | 113.79 (99.43,130.01) | 7576 (6603,8611) | 113.98 (99.53,129.49) | 0.01 (-0.01,0.02) | 0.03 |
| Lithuania | 5078 (4393,5849) | 111.58 (97.39,128.02) | 7771 (6694,9018) | 112.73 (98.22,129.2) | 0.04 (0.01,0.06) | 0.02 |
| Lebanon | 2570 (2255,2921) | 140.43 (123.12,159.83) | 9167 (8003,10453) | 140.48 (122.53,159.62) | -0.01 (-0.02,0) | 0.02 |
| Mauritius | 660 (569,758) | 112.09 (97.53,128.76) | 1883 (1629,2139) | 111.73 (96.55,127.55) | 0.03 (0,0.05) | 0.02 |
| Hungary | 15846 (13689,18464) | 112.25 (98.26,128.61) | 24331 (21065,27869) | 111.47 (96.69,127.63) | -0.03 (-0.06,0.01) | 0.02 |
| Armenia | 2585 (2259,2947) | 114.47 (99.79,130.46) | 4930 (4260,5655) | 114 (99.56,130.25) | -0.02 (-0.03,-0.01) | 0.01 |
| Solomon Islands | 97 (83,111) | 112.84 (98.27,129.48) | 256 (219,295) | 113.74 (98.07,131.08) | 0.07 (0.05,0.09) | 0.01 |
| Uzbekistan | 11398 (9888,13130) | 106.85 (93,122.73) | 20088 (17467,22885) | 106.19 (92.22,122.08) | -0.03 (-0.05,-0.02) | 0.01 |
| Belarus | 14456 (12531,16564) | 115.82 (100.91,132.62) | 19115 (16467,21917) | 116.35 (101.03,133.43) | 0.02 (0,0.03) | 0.00 |
| Nicaragua | 1601 (1403,1813) | 114.78 (100.41,130.39) | 4883 (4277,5562) | 114.62 (100.58,131.05) | 0.01 (0,0.02) | 0.00 |
| Guatemala | 2921 (2552,3331) | 113.43 (99.15,129.47) | 10938 (9620,12428) | 112.54 (98.73,127.7) | -0.02 (-0.03,0) | -0.01 |
| Brunei Darussalam | 81 (70,94) | 101.78 (87.9,117.59) | 237 (205,273) | 101.56 (88.37,116.86) | 0.04 (0,0.08) | -0.01 |
| Colombia | 17564 (15423,19922) | 113.29 (98.82,129.37) | 63668 (55802,72058) | 112.13 (97.75,127.51) | -0.04 (-0.06,-0.02) | -0.01 |
| Brazil | 98068 (85802,111118) | 129.04 (113.25,146.32) | 305421 (269292,345696) | 127.08 (112.01,144.66) | -0.13 (-0.17,-0.09) | -0.01 |
| North Macedonia | 1748 (1524,1991) | 111.77 (97.43,128.12) | 3033 (2587,3532) | 111.12 (96.41,127.65) | 0 (-0.01,0.01) | -0.01 |
| Russian Federation | 190282 (164523,219606) | 117.56 (102.69,134.45) | 279806 (243531,319869) | 115.89 (101.23,131.98) | -0.1 (-0.15,-0.06) | -0.01 |
| Portugal | 16587 (14199,19312) | 118.79 (102.75,136.06) | 35666 (30670,41514) | 116.97 (100.45,134.76) | -0.03 (-0.05,-0.01) | -0.01 |
| Romania | 27277 (23253,31597) | 111.59 (96.91,127.8) | 46175 (39788,53464) | 111.32 (96.82,127.73) | -0.04 (-0.06,-0.03) | -0.02 |
| Saint Kitts and Nevis | 35 (30,41) | 94.4 (82.44,108.43) | 48 (42,56) | 93.38 (80.85,107.47) | -0.05 (-0.06,-0.04) | -0.02 |
| Bolivia (Plurinational State of) | 2131 (1841,2439) | 82.56 (71.74,94.39) | 6052 (5222,6916) | 82.05 (71.22,94.16) | 0.01 (-0.01,0.03) | -0.02 |
| Guyana | 292 (254,332) | 97.25 (83.98,111.33) | 481 (419,548) | 96.25 (83.56,110.32) | -0.05 (-0.06,-0.04) | -0.02 |
| Malawi | 2832 (2442,3230) | 105.4 (91.67,120.77) | 5280 (4599,6027) | 104.71 (90.82,119.89) | -0.01 (-0.03,0.01) | -0.02 |
| Czechia | 15203 (13105,17550) | 112.36 (97.87,128.36) | 26327 (22779,30305) | 111.23 (96.31,127.59) | -0.06 (-0.07,-0.05) | -0.02 |
| Democratic Republic of the Congo | 13164 (11342,15031) | 126.78 (110.3,143.99) | 29847 (26225,33722) | 126.99 (111.77,144.57) | 0.01 (-0.03,0.04) | -0.03 |
| Kazakhstan | 11933 (10475,13665) | 113.6 (99.49,130.25) | 15444 (13387,17642) | 111.1 (96.76,126.81) | -0.09 (-0.11,-0.06) | -0.03 |
| Northern Mariana Islands | 11 (9,13) | 109.43 (93.88,127.66) | 38 (32,44) | 107.94 (92.46,125.17) | -0.03 (-0.05,-0.02) | -0.03 |
| Uganda | 5022 (4361,5706) | 104.06 (90.86,118.38) | 10731 (9352,12174) | 102.68 (89.05,117.37) | -0.06 (-0.08,-0.04) | -0.03 |
| Somalia | 1509 (1313,1697) | 107.43 (93.77,122.3) | 3590 (3086,4105) | 106.72 (93.34,121.51) | -0.01 (-0.02,0) | -0.03 |
| Panama | 1607 (1404,1825) | 110.15 (96,125.54) | 4943 (4336,5638) | 108.64 (94.58,124.54) | -0.05 (-0.06,-0.05) | -0.04 |
| Guam | 60 (51,70) | 109.68 (94.45,127.52) | 242 (210,275) | 108.97 (93.85,125.54) | -0.03 (-0.04,-0.02) | -0.04 |
| Lao People's Democratic Republic | 1660 (1430,1896) | 113.69 (99.88,129.87) | 3764 (3280,4290) | 111.82 (97.43,127.89) | -0.05 (-0.06,-0.03) | -0.04 |
| Bermuda | 59 (50,68) | 99.71 (86.05,114.57) | 150 (130,173) | 98.24 (84.8,112.71) | -0.06 (-0.06,-0.05) | -0.04 |
| Chile | 10283 (9008,11672) | 109.81 (96.22,123.58) | 28466 (24777,32535) | 107.87 (93.91,123.11) | -0.03 (-0.05,0) | -0.04 |
| Greenland | 32 (27,36) | 139.66 (121.08,159.9) | 69 (60,79) | 135.63 (117.63,156.11) | -0.09 (-0.11,-0.07) | -0.04 |
| Qatar | 81 (70,91) | 128.19 (111.46,146.56) | 640 (553,721) | 126.56 (110.1,145.4) | 0.02 (-0.02,0.05) | -0.05 |
| Ecuador | 4011 (3471,4605) | 82.43 (71.41,94.77) | 12517 (10756,14473) | 81.16 (69.99,93.4) | -0.02 (-0.04,-0.01) | -0.05 |
| Honduras | 2026 (1771,2313) | 114.77 (100.53,131.28) | 5675 (4919,6495) | 113.19 (98.75,129.15) | -0.05 (-0.06,-0.05) | -0.05 |
| Trinidad and Tobago | 786 (682,904) | 100.44 (88.01,114.8) | 1821 (1586,2070) | 98.6 (85.73,112.51) | -0.06 (-0.06,-0.05) | -0.05 |
| Slovakia | 6446 (5589,7369) | 111.96 (97.71,127.76) | 10646 (9281,12234) | 110.87 (97.08,127) | -0.05 (-0.06,-0.04) | -0.05 |
| Afghanistan | 6914 (5989,7884) | 132.98 (116.42,151.73) | 8764 (7688,10026) | 130.94 (115.12,149.09) | -0.06 (-0.07,-0.04) | -0.05 |
| Bahamas | 136 (119,156) | 97.08 (84.51,110.72) | 326 (285,372) | 95.28 (82.97,109.64) | -0.08 (-0.08,-0.07) | -0.05 |
| Tokelau | 1 (1,2) | 111.41 (96.19,128.32) | 2 (1,2) | 109.37 (94.11,126.6) | -0.06 (-0.07,-0.05) | -0.05 |
| Cook Islands | 11 (10,13) | 111.77 (95.49,129.15) | 28 (24,32) | 109.53 (93.6,126.15) | -0.08 (-0.08,-0.07) | -0.06 |
| Tuvalu | 5 (5,6) | 115.76 (100.06,132.92) | 9 (8,11) | 113.41 (97.97,130.03) | -0.06 (-0.07,-0.05) | -0.06 |
| Lesotho | 769 (669,876) | 109.88 (95.45,125.25) | 791 (682,906) | 108.92 (94.83,124.74) | -0.01 (-0.02,0) | -0.06 |
| Venezuela (Bolivarian Republic of) | 11339 (9902,12877) | 128.24 (111.91,145.3) | 34325 (30254,38771) | 124.37 (108.4,141.62) | -0.11 (-0.14,-0.07) | -0.06 |
| Croatia | 6148 (5344,7085) | 115.77 (101.1,133.22) | 11299 (9711,12993) | 112.67 (98.23,128.39) | -0.07 (-0.08,-0.06) | -0.06 |
| Peru | 8933 (7783,10125) | 79.62 (69.34,90.7) | 25917 (22445,29526) | 78.28 (67.57,89.77) | -0.11 (-0.13,-0.09) | -0.06 |
| Grenada | 83 (72,96) | 99.56 (86.98,114.23) | 92 (79,105) | 98.02 (85.92,112.22) | -0.03 (-0.06,0) | -0.06 |
| Iran (Islamic Republic of) | 25143 (21708,28637) | 137.09 (120.6,155.91) | 87937 (77508,99879) | 133.73 (117.53,151.85) | -0.07 (-0.08,-0.07) | -0.06 |
| Costa Rica | 1972 (1721,2256) | 113.01 (98.53,129.41) | 6143 (5378,6985) | 110.62 (95.96,126.87) | -0.08 (-0.09,-0.06) | -0.06 |
| American Samoa | 18 (15,21) | 111.48 (96.23,128.37) | 41 (35,47) | 109.14 (93.39,125.47) | -0.09 (-0.09,-0.08) | -0.06 |
| Vanuatu | 44 (37,51) | 107.72 (93.33,123.98) | 119 (101,138) | 105.3 (90.64,121.61) | -0.1 (-0.12,-0.08) | -0.07 |
| Bulgaria | 12362 (10399,14581) | 115.07 (100.11,132.17) | 17299 (14848,20198) | 112.43 (97.8,128.73) | -0.1 (-0.12,-0.07) | -0.07 |
| Antigua and Barbuda | 58 (50,67) | 97.18 (84.42,111.33) | 88 (76,101) | 95.08 (82.7,109.64) | -0.06 (-0.08,-0.05) | -0.07 |
| Indonesia | 84100 (73439,95482) | 115.95 (101.29,132.28) | 189722 (164142,216604) | 113.31 (98.38,129.52) | -0.08 (-0.1,-0.06) | -0.07 |
| Angola | 3105 (2700,3510) | 127.18 (111.54,144.47) | 9171 (7979,10423) | 124.21 (109.07,141.23) | -0.08 (-0.08,-0.07) | -0.07 |
| Italy | 111576 (94488,128892) | 115.83 (98.69,132.49) | 248809 (215069,285482) | 134.76 (116.74,153.65) | 0.32 (0.12,0.53) | -0.07 |
| Ukraine | 78566 (67889,90270) | 119.02 (103.91,135.64) | 93467 (80738,107940) | 115.57 (100.64,132.68) | -0.13 (-0.17,-0.09) | -0.07 |
| Marshall Islands | 12 (10,14) | 107.18 (92.38,123.7) | 21 (17,24) | 103.94 (89.43,120.5) | -0.1 (-0.11,-0.1) | -0.07 |
| Bahrain | 144 (124,164) | 133.15 (116.31,151.71) | 692 (594,788) | 130.61 (114.78,148.7) | -0.04 (-0.05,-0.03) | -0.07 |
| Ghana | 3390 (2934,3845) | 76.62 (66,88) | 8608 (7446,9814) | 75.09 (64.72,86.42) | -0.05 (-0.06,-0.04) | -0.07 |
| Palau | 8 (7,10) | 106.89 (91.51,124.12) | 16 (13,18) | 103.98 (88.42,120.53) | -0.09 (-0.09,-0.08) | -0.08 |
| Montenegro | 682 (593,782) | 114.96 (100.51,131.88) | 1006 (863,1163) | 111.66 (97.13,128.15) | -0.15 (-0.19,-0.11) | -0.08 |
| Azerbaijan | 4820 (4212,5530) | 113.7 (99.47,130.16) | 8311 (7218,9441) | 110.27 (96.65,126.18) | -0.14 (-0.16,-0.12) | -0.08 |
| Gabon | 586 (509,669) | 125.64 (109.37,143.64) | 911 (794,1042) | 123.01 (107.72,140.92) | -0.06 (-0.07,-0.04) | -0.08 |
| Fiji | 266 (225,307) | 112.81 (97.29,130.02) | 582 (495,672) | 110.65 (95.5,127.91) | -0.09 (-0.1,-0.07) | -0.08 |
| Zimbabwe | 3214 (2773,3675) | 105.39 (91.76,120.16) | 4505 (3921,5133) | 102.44 (89.41,116.93) | -0.1 (-0.11,-0.09) | -0.08 |
| Puerto Rico | 3779 (3263,4354) | 99.76 (87.12,114.02) | 8775 (7670,10036) | 97.8 (84.88,112.14) | -0.08 (-0.09,-0.08) | -0.08 |
| Namibia | 489 (422,556) | 106.73 (93.53,121.78) | 1012 (884,1148) | 103.46 (89.84,117.7) | -0.1 (-0.12,-0.09) | -0.08 |
| Saint Lucia | 81 (69,94) | 99.01 (86.18,113.38) | 223 (192,255) | 95.98 (82.99,109.74) | -0.14 (-0.16,-0.11) | -0.08 |
| Egypt | 23384 (20531,26090) | 128.38 (114.25,144.51) | 50931 (44459,57309) | 126.46 (111.45,144.07) | -0.03 (-0.05,0) | -0.08 |
| Kenya | 7010 (6119,7949) | 107.07 (93.41,121.96) | 16494 (14435,18732) | 105.14 (91.55,119.62) | -0.07 (-0.09,-0.05) | -0.08 |
| Samoa | 73 (62,84) | 111.57 (96.41,128.78) | 125 (107,144) | 108.37 (93.13,125.19) | -0.11 (-0.12,-0.11) | -0.09 |
| Guinea-Bissau | 203 (174,231) | 78.72 (67.95,90.63) | 330 (283,376) | 77.05 (66.66,89.04) | -0.06 (-0.07,-0.05) | -0.09 |
| Comoros | 147 (127,168) | 104.63 (91.44,118.88) | 381 (330,432) | 100.96 (88.05,115.39) | -0.11 (-0.12,-0.1) | -0.09 |
| Malaysia | 9527 (8380,10775) | 116.29 (101.79,132.63) | 26046 (22443,29858) | 111.29 (95.99,127.46) | -0.13 (-0.15,-0.11) | -0.09 |
| Niue | 3 (2,3) | 110.15 (95.02,127.07) | 2 (2,3) | 107.28 (91.33,123.83) | -0.11 (-0.12,-0.09) | -0.09 |
| Central African Republic | 923 (791,1047) | 134.26 (118.33,152.43) | 1622 (1391,1837) | 130.87 (114.21,148.33) | -0.11 (-0.12,-0.11) | -0.09 |
| Democratic People's Republic of Korea | 13116 (11272,15096) | 110.97 (95.88,127.95) | 31408 (26865,36668) | 108.5 (93.89,125.37) | -0.1 (-0.12,-0.08) | -0.09 |
| Equatorial Guinea | 167 (146,190) | 129.07 (112.91,146.07) | 431 (377,490) | 124.45 (108.79,141.57) | -0.15 (-0.16,-0.14) | -0.09 |
| Sweden | 24442 (21503,27653) | 134.31 (118.97,151.02) | 33511 (29061,38427) | 126.32 (109.55,144.06) | -0.2 (-0.23,-0.18) | -0.10 |
| Congo | 908 (783,1044) | 124.09 (108.43,141.67) | 2079 (1886,2269) | 120.8 (109.94,132.53) | -0.09 (-0.11,-0.06) | -0.10 |
| Tonga | 52 (45,60) | 115.12 (100.05,132.42) | 84 (73,97) | 111.8 (97.12,128.75) | -0.11 (-0.13,-0.1) | -0.10 |
| Botswana | 424 (365,483) | 107.65 (93.62,122.91) | 1062 (924,1218) | 103.94 (90.71,119.61) | -0.1 (-0.13,-0.08) | -0.10 |
| Cyprus | 878 (744,1037) | 121.53 (105.52,138.93) | 2459 (2096,2871) | 117.37 (101.52,134.87) | -0.09 (-0.1,-0.07) | -0.10 |
| Côte d'Ivoire | 2044 (1770,2322) | 79.52 (68.92,91.37) | 5598 (4850,6401) | 76.63 (66.06,88.28) | -0.14 (-0.15,-0.12) | -0.10 |
| Belize | 95 (83,109) | 100.29 (87.44,114.78) | 244 (214,276) | 97.17 (84.64,110.79) | -0.11 (-0.11,-0.1) | -0.10 |
| Dominica | 59 (51,68) | 99.29 (86.4,113.22) | 74 (64,84) | 96.57 (83.47,110.4) | -0.09 (-0.1,-0.09) | -0.11 |
| Liberia | 694 (596,796) | 77.6 (67.38,88.97) | 1101 (956,1253) | 75.16 (64.95,86.14) | -0.11 (-0.13,-0.1) | -0.11 |
| Barbados | 330 (285,385) | 98.36 (86.39,112.47) | 497 (433,573) | 94.55 (82.58,109.16) | -0.15 (-0.17,-0.13) | -0.11 |
| Togo | 709 (619,805) | 80.67 (69.92,92.56) | 1900 (1634,2158) | 78.26 (67.73,90) | -0.09 (-0.1,-0.08) | -0.11 |
| Dominican Republic | 3358 (2922,3844) | 100 (87.38,113.63) | 9479 (8275,10748) | 99.53 (86.68,113.07) | -0.15 (-0.18,-0.11) | -0.11 |
| Djibouti | 94 (82,107) | 108.76 (95.11,123.96) | 402 (347,454) | 104.02 (90.57,118.65) | -0.12 (-0.13,-0.1) | -0.11 |
| Mozambique | 4509 (3902,5110) | 107.36 (94.05,122.84) | 7516 (6505,8549) | 104.33 (90.49,119.04) | -0.08 (-0.1,-0.07) | -0.12 |
| Seychelles | 64 (56,74) | 113.24 (98.52,129.83) | 108 (94,124) | 109.25 (94.47,126.6) | -0.14 (-0.14,-0.13) | -0.12 |
| United States Virgin Islands | 69 (59,79) | 97.13 (84.11,111.29) | 181 (156,212) | 94.57 (82.02,109.17) | -0.11 (-0.12,-0.1) | -0.12 |
| Mali | 2169 (1879,2470) | 81.5 (71.35,93.08) | 4712 (4072,5355) | 78.55 (68.4,89.84) | -0.12 (-0.13,-0.12) | -0.12 |
| Eswatini | 212 (184,241) | 104.32 (90.99,119.37) | 359 (310,411) | 101.07 (88.65,115.8) | -0.12 (-0.13,-0.11) | -0.12 |
| Bangladesh | 30924 (26840,35296) | 82.3 (70.98,94.25) | 92254 (79987,105807) | 79.47 (68.72,90.87) | -0.12 (-0.13,-0.11) | -0.12 |
| Palestine | 983 (859,1118) | 136.85 (120.04,155.15) | 2402 (2103,2729) | 131.55 (115.83,150.08) | -0.15 (-0.17,-0.13) | -0.13 |
| United States of America | 492880 (429701,564818) | 138.35 (120.88,157.03) | 822911 (718803,935112) | 131.29 (113.93,149.6) | -0.19 (-0.21,-0.18) | -0.13 |
| Cambodia | 3785 (3277,4314) | 116.54 (101.62,132.77) | 10162 (8838,11662) | 112.21 (97.73,128.37) | -0.18 (-0.19,-0.16) | -0.13 |
| Kuwait | 603 (530,678) | 136.6 (119.53,155.03) | 2814 (2484,3155) | 131.46 (115.44,149.34) | -0.14 (-0.16,-0.12) | -0.13 |
| Greece | 19618 (16834,22921) | 124.1 (107.53,142.61) | 37087 (31838,42954) | 119.17 (103.16,136.72) | -0.12 (-0.14,-0.09) | -0.13 |
| Algeria | 13226 (11355,15365) | 133.66 (116.97,152.44) | 37174 (32167,43066) | 128.59 (112.65,146.91) | -0.14 (-0.15,-0.14) | -0.14 |
| Sri Lanka | 9699 (8465,11058) | 110.9 (96.53,126.63) | 25786 (22299,29882) | 107.46 (93.3,123.54) | -0.12 (-0.14,-0.11) | -0.14 |
| Eritrea | 611 (526,692) | 109.92 (95.74,125.59) | 1735 (1503,1975) | 104.21 (90.44,119.18) | -0.18 (-0.18,-0.17) | -0.14 |
| Cuba | 9728 (8493,11029) | 93.06 (81.15,105.22) | 19278 (16865,21941) | 91.41 (79.7,103.65) | -0.23 (-0.27,-0.19) | -0.15 |
| South Sudan | 2028 (1756,2318) | 104.61 (91.25,119.47) | 2565 (2229,2899) | 99.16 (85.85,113.38) | -0.17 (-0.19,-0.14) | -0.15 |
| Madagascar | 3808 (3315,4320) | 104.43 (91.35,119.44) | 6743 (5808,7654) | 99.76 (86.8,114.13) | -0.14 (-0.16,-0.13) | -0.15 |
| South Africa | 20560 (17955,23217) | 113.1 (98.7,129.16) | 39715 (34529,45264) | 107.78 (93.63,122.59) | -0.14 (-0.15,-0.13) | -0.15 |
| Turkmenistan | 1690 (1483,1919) | 112.46 (98.71,127.9) | 3296 (2869,3741) | 107.05 (93.39,122.99) | -0.2 (-0.22,-0.17) | -0.15 |
| Suriname | 240 (209,272) | 104.75 (91.6,119.86) | 580 (506,663) | 100.19 (87.41,115.47) | -0.17 (-0.19,-0.16) | -0.15 |
| France | 94247 (84334,103887) | 98.87 (89.02,109) | 163141 (143062,184345) | 93.63 (82.25,105.56) | -0.22 (-0.26,-0.18) | -0.16 |
| Saint Vincent and the Grenadines | 69 (60,80) | 101 (87.52,115.23) | 128 (111,146) | 96.56 (84.19,110.27) | -0.16 (-0.17,-0.16) | -0.16 |
| India | 257735 (224227,293550) | 79.04 (68.33,90.3) | 749490 (648646,856823) | 78.92 (68.29,90.58) | -0.15 (-0.2,-0.1) | -0.16 |
| Poland | 49371 (42659,56817) | 120.12 (104.53,137) | 88407 (76685,101187) | 114.27 (99.44,130.4) | -0.17 (-0.19,-0.16) | -0.16 |
| United Kingdom | 115427 (99727,133198) | 113.91 (99.28,130.08) | 164947 (142721,190062) | 107.93 (93.37,123.89) | -0.17 (-0.21,-0.13) | -0.16 |
| Iraq | 9666 (8465,10949) | 135.31 (118.13,154.01) | 22204 (19382,25184) | 129.62 (113.05,148.98) | -0.18 (-0.19,-0.17) | -0.16 |
| Niger | 1526 (1319,1732) | 82.8 (71.98,95.34) | 4296 (3712,4924) | 78.36 (68.05,90.06) | -0.2 (-0.2,-0.19) | -0.17 |
| Tajikistan | 2626 (2296,2978) | 111.49 (97.32,126.93) | 4157 (3626,4715) | 104.98 (92.03,120.4) | -0.23 (-0.24,-0.22) | -0.17 |
| Sierra Leone | 1391 (1208,1594) | 83.45 (72.82,94.85) | 2201 (1908,2531) | 79.15 (68.54,91.02) | -0.19 (-0.2,-0.17) | -0.18 |
| Libya | 2264 (1997,2549) | 136.08 (119.46,154.53) | 5287 (4619,5998) | 130.47 (114.04,148.13) | -0.14 (-0.16,-0.13) | -0.18 |
| Philippines | 27813 (24209,31882) | 118.99 (104.75,135.44) | 72092 (63052,81980) | 113.91 (99.74,130) | -0.19 (-0.21,-0.17) | -0.18 |
| Ethiopia | 13750 (11888,15616) | 109.29 (95.57,124.26) | 34226 (30050,38961) | 102.87 (90.07,116.73) | -0.18 (-0.21,-0.15) | -0.18 |
| Argentina | 34827 (30211,39910) | 112.03 (97.43,128.19) | 62825 (54326,72188) | 106.73 (92.2,122.29) | -0.18 (-0.19,-0.16) | -0.18 |
| Cameroon | 2565 (2220,2919) | 79.31 (68.75,90.99) | 6433 (5588,7316) | 75.45 (65.39,86.37) | -0.18 (-0.18,-0.17) | -0.18 |
| Saudi Arabia | 5686 (4959,6481) | 127.56 (111.24,146.12) | 13064 (11315,14779) | 120.44 (104.42,138.45) | -0.19 (-0.21,-0.18) | -0.19 |
| Mauritania | 652 (559,745) | 82.98 (71.62,95.11) | 1316 (1142,1500) | 77.53 (67.33,88.83) | -0.24 (-0.24,-0.23) | -0.19 |
| Oman | 666 (582,753) | 131.81 (115.59,150.61) | 1619 (1401,1863) | 124.36 (108.14,143.21) | -0.17 (-0.22,-0.13) | -0.19 |
| New Zealand | 5268 (4565,6055) | 127.8 (111.1,145.96) | 11097 (9639,12753) | 120.84 (104.92,138.57) | -0.24 (-0.27,-0.21) | -0.19 |
| Sao Tome and Principe | 42 (36,48) | 78.02 (67.66,89.88) | 60 (52,70) | 73.4 (63.08,84.79) | -0.19 (-0.21,-0.17) | -0.19 |
| Jamaica | 2182 (1901,2498) | 110.25 (96.1,125.59) | 3490 (3046,3974) | 104.77 (91.01,120.07) | -0.21 (-0.22,-0.19) | -0.19 |
| Timor-Leste | 223 (195,251) | 119.3 (103.86,136.18) | 768 (666,888) | 113.2 (98.46,129.74) | -0.17 (-0.19,-0.15) | -0.19 |
| Paraguay | 2629 (2303,2975) | 122.93 (107.39,139.55) | 6135 (5388,6970) | 115.77 (101.31,132.33) | -0.23 (-0.24,-0.22) | -0.19 |
| Türkiye | 42666 (37375,48311) | 147.16 (128.67,166.63) | 117991 (102838,133396) | 139.1 (120.57,158.16) | -0.23 (-0.25,-0.22) | -0.20 |
| Uruguay | 4635 (4017,5293) | 113.1 (98.89,128.49) | 7017 (6124,8053) | 107.09 (93.07,121.78) | -0.23 (-0.25,-0.21) | -0.20 |
| Cabo Verde | 209 (180,242) | 81.92 (70.75,93.85) | 319 (276,363) | 78.13 (67.85,89.4) | -0.18 (-0.18,-0.17) | -0.20 |
| Tunisia | 6004 (5202,6825) | 141.66 (124.04,160.74) | 15845 (13951,18011) | 134.32 (117.99,153.04) | -0.18 (-0.19,-0.18) | -0.20 |
| Viet Nam | 41585 (36380,47477) | 116.55 (102.43,133.75) | 90172 (78824,102840) | 110.09 (96.21,126.57) | -0.23 (-0.24,-0.22) | -0.21 |
| Guinea | 2309 (2006,2617) | 83.04 (72.5,94.9) | 3372 (2920,3850) | 78.39 (67.86,90.01) | -0.2 (-0.22,-0.19) | -0.21 |
| Andorra | 71 (60,81) | 121.02 (104.88,138.58) | 190 (164,218) | 114.28 (98.41,131.55) | -0.2 (-0.21,-0.19) | -0.21 |
| Syrian Arab Republic | 5648 (4959,6393) | 138.62 (121.31,157.84) | 13051 (11335,14936) | 129.77 (113.5,147.95) | -0.24 (-0.24,-0.23) | -0.22 |
| Austria | 16276 (14023,18889) | 123.65 (107.51,141.53) | 25291 (21751,29369) | 116.09 (99.55,133.87) | -0.26 (-0.29,-0.24) | -0.22 |
| Mexico | 37949 (32951,43436) | 103.1 (89.53,117.63) | 110467 (96168,125494) | 97.19 (84.27,111.26) | -0.14 (-0.17,-0.12) | -0.22 |
| United Arab Emirates | 345 (297,398) | 123.25 (107.14,142.51) | 2324 (1963,2691) | 113.49 (98.05,131.08) | -0.27 (-0.31,-0.24) | -0.22 |
| Sudan | 10295 (8913,11733) | 133.97 (117.29,152.94) | 18538 (16236,21018) | 125.81 (110.08,143.27) | -0.23 (-0.24,-0.22) | -0.22 |
| Israel | 6189 (5277,7208) | 119.68 (103.31,137.43) | 15408 (13247,17722) | 113.13 (97.01,130.26) | -0.21 (-0.21,-0.2) | -0.23 |
| United Republic of Tanzania | 8601 (7577,9681) | 105.95 (93.42,119.2) | 18873 (16815,21095) | 98.48 (87.78,110.14) | -0.24 (-0.26,-0.23) | -0.23 |
| Papua New Guinea | 1332 (1143,1512) | 119.96 (105.09,137.12) | 3500 (3026,3981) | 112.89 (97.86,129.7) | -0.27 (-0.29,-0.24) | -0.23 |
| Burundi | 1863 (1608,2126) | 108.15 (94.65,122.98) | 3237 (2823,3672) | 100.5 (88.07,114.87) | -0.22 (-0.24,-0.21) | -0.23 |
| Senegal | 2055 (1771,2337) | 82.01 (71.31,94) | 4513 (3919,5167) | 76.64 (66.53,88.26) | -0.26 (-0.27,-0.25) | -0.23 |
| Burkina Faso | 2714 (2348,3111) | 84.91 (73.58,97.02) | 5394 (4695,6140) | 79.8 (69.33,91.77) | -0.23 (-0.24,-0.23) | -0.23 |
| Pakistan | 39180 (33966,44720) | 84.21 (73.14,96.59) | 67818 (58773,77287) | 78.8 (67.99,90.38) | -0.25 (-0.26,-0.25) | -0.23 |
| Gambia | 216 (188,247) | 83.67 (72.87,95.98) | 573 (493,658) | 77.92 (67.57,89.88) | -0.25 (-0.26,-0.25) | -0.23 |
| Bhutan | 138 (120,157) | 83.15 (72.17,95.37) | 415 (360,475) | 77.45 (67.36,88.68) | -0.24 (-0.26,-0.21) | -0.23 |
| Morocco | 17075 (14858,19361) | 136.09 (118.85,155.06) | 36514 (31637,41831) | 127.85 (111.31,146.15) | -0.23 (-0.23,-0.22) | -0.23 |
| Haiti | 2321 (2000,2642) | 101.59 (88.48,115.53) | 4672 (4066,5354) | 94.82 (82.42,108.66) | -0.26 (-0.28,-0.24) | -0.23 |
| Myanmar | 20696 (17941,23619) | 121.36 (106.48,137.66) | 44028 (38392,49952) | 113.09 (99.06,128.82) | -0.26 (-0.28,-0.25) | -0.25 |
| Malta | 515 (443,596) | 122.04 (105.66,140) | 1295 (1113,1493) | 113.91 (98.03,130.83) | -0.21 (-0.23,-0.19) | -0.25 |
| Yemen | 5035 (4373,5725) | 143.07 (125.88,162.62) | 13410 (11724,15191) | 133.11 (117.14,151.66) | -0.26 (-0.28,-0.23) | -0.25 |
| Ireland | 5187 (4445,6032) | 122.19 (105.76,140.41) | 9579 (8205,11080) | 111.99 (95.75,128.99) | -0.31 (-0.33,-0.29) | -0.25 |
| Nigeria | 25694 (22282,29177) | 75.1 (65.04,85.62) | 45312 (39635,51186) | 68.83 (59.58,78.6) | -0.3 (-0.34,-0.25) | -0.26 |
| Benin | 1376 (1208,1566) | 81.84 (71.81,93.16) | 2909 (2552,3316) | 75.85 (65.91,87.05) | -0.2 (-0.22,-0.18) | -0.27 |
| Monaco | 108 (93,126) | 122.71 (106.16,141.01) | 136 (116,157) | 113.72 (97.81,131.33) | -0.28 (-0.29,-0.27) | -0.27 |
| Chad | 1931 (1675,2212) | 83.52 (72.31,95.78) | 3052 (2649,3472) | 76.45 (66.54,87.28) | -0.32 (-0.33,-0.31) | -0.30 |
| Spain | 66079 (57996,73532) | 112.03 (98.68,123.28) | 123770 (107148,142152) | 102.5 (88.99,116.45) | -0.32 (-0.39,-0.25) | -0.31 |
| Finland | 9390 (8168,10732) | 120.98 (105.76,137.25) | 16662 (14213,19329) | 108.45 (92.52,124.81) | -0.41 (-0.42,-0.4) | -0.32 |
| Switzerland | 15095 (13093,17295) | 124.5 (108.87,142.44) | 24776 (21498,28441) | 113.27 (97.88,129.61) | -0.35 (-0.38,-0.33) | -0.34 |
| Iceland | 429 (375,485) | 134.35 (117.61,151.24) | 799 (689,898) | 122.36 (106.34,137.53) | -0.37 (-0.39,-0.35) | -0.35 |
| Belgium | 23042 (20167,26295) | 136.46 (120.43,154.4) | 34245 (29719,39179) | 121.59 (105.24,139.55) | -0.45 (-0.47,-0.42) | -0.36 |
| San Marino | 52 (45,59) | 123.67 (108.13,141.19) | 106 (92,122) | 110.9 (96.02,127.71) | -0.39 (-0.41,-0.37) | -0.39 |
| Nepal | 6014 (5254,6820) | 90.94 (79.26,103.61) | 14856 (12919,16960) | 81.31 (70.51,93.7) | -0.47 (-0.5,-0.44) | -0.40 |
| Canada | 53906 (48456,59760) | 155.64 (140.55,171.87) | 105264 (93819,117160) | 132.4 (118.02,147.29) | -0.62 (-0.65,-0.58) | -0.40 |
| Australia | 24675 (21656,27883) | 120.3 (106.3,134.67) | 52191 (45954,58772) | 102.66 (90.27,115.36) | -0.62 (-0.68,-0.57) | -0.44 |
| Luxembourg | 578 (491,675) | 100.97 (86.52,116.93) | 1033 (880,1191) | 87.36 (74.31,101.13) | -0.5 (-0.57,-0.43) | -0.45 |
| Norway | 10767 (9395,12373) | 134.49 (117.52,152.85) | 13149 (11352,15113) | 114.3 (98.69,131.41) | -0.64 (-0.69,-0.6) | -0.58 |
| Denmark | 9424 (8279,10766) | 102.16 (90.11,115.97) | 11466 (9906,13148) | 84.34 (72.78,96.21) | -0.73 (-0.77,-0.69) | -0.68 |

**S2 Table.** APC analysis of ADRD incidence in global and five SDI regions across sex from 1992 to 2021.

| **Sex** | **Location** | **WaldTests** | **X2** | **df** | **P-Value** |
| --- | --- | --- | --- | --- | --- |
| **Both** | **Global** | NetDrift = 0 | 2.87655730480962 | 1 | 0.08 |
|  |  | All Period RR = 1 | 37.2622568620691 | 5 | <0.001 |
|  |  | All Cohort RR = 1 | 453.050773724016 | 16 | <0.001 |
|  |  | All Local Drifts = Net Drift | 439.734073395407 | 12 | <0.001 |
|  | **Low SDI** | NetDrift = 0 | 237.6905390028 | 1 | <0.001 |
|  |  | All Period RR = 1 | 241.031263822595 | 5 | <0.001 |
|  |  | All Cohort RR = 1 | 418.271860609472 | 16 | <0.001 |
|  |  | All Local Drifts = Net Drift | 37.8475476623434 | 12 | <0.001 |
|  | **Low-middle SDI** | NetDrift = 0 | 370.748411108647 | 1 | <0.001 |
|  |  | All Period RR = 1 | 395.158298738538 | 5 | <0.001 |
|  |  | All Cohort RR = 1 | 911.365244212795 | 16 | <0.001 |
|  |  | All Local Drifts = Net Drift | 142.224534263594 | 12 | <0.001 |
|  | **Middle SDI** | NetDrift = 0 | 23.9047858700334 | 1 | <0.001 |
|  |  | All Period RR = 1 | 101.592736856353 | 5 | <0.001 |
|  |  | All Cohort RR = 1 | 159.157228102951 | 16 | <0.001 |
|  |  | All Local Drifts = Net Drift | 99.339230422065 | 12 | <0.001 |
|  | **High-middle SDI** | NetDrift = 0 | 147.165631398448 | 1 | <0.001 |
|  |  | All Period RR = 1 | 237.75432493495 | 5 | <0.001 |
|  |  | All Cohort RR = 1 | 327.638974878376 | 16 | <0.001 |
|  |  | All Local Drifts = Net Drift | 43.9351797611831 | 12 | <0.001 |
|  | **High SDI** | NetDrift = 0 | 0.00834304489628639 | 1 | 0.927 |
|  |  | All Period RR = 1 | 53.9367085697137 | 5 | <0.001 |
|  |  | All Cohort RR = 1 | 539.419630389002 | 16 | <0.001 |
|  |  | All Local Drifts = Net Drift | 409.057394060979 | 12 | <0.001 |
| **Female** | **Global** | NetDrift = 0 | 6.87983848613402 | 1 | 0.009 |
|  |  | All Period RR = 1 | 61.9954983716895 | 5 | <0.001 |
|  |  | All Cohort RR = 1 | 452.242289387115 | 16 | <0.001 |
|  |  | All Local Drifts = Net Drift | 446.465090431047 | 12 | <0.001 |
|  | **Low SDI** | NetDrift = 0 | 170.300135452701 | 1 | <0.001 |
|  |  | All Period RR = 1 | 172.826948391881 | 5 | <0.001 |
|  |  | All Cohort RR = 1 | 344.074879981023 | 16 | <0.001 |
|  |  | All Local Drifts = Net Drift | 51.0703005353117 | 12 | <0.001 |
|  | **Low-middle SDI** | NetDrift = 0 | 447.485468164719 | 1 | <0.001 |
|  |  | All Period RR = 1 | 473.636219466745 | 5 | <0.001 |
|  |  | All Cohort RR = 1 | 960.950625150521 | 16 | <0.001 |
|  |  | All Local Drifts = Net Drift | 91.1623106194425 | 12 | <0.001 |
|  | **Middle SDI** | NetDrift = 0 | 4.75764109459555 | 1 | 0.029 |
|  |  | All Period RR = 1 | 71.8366124315351 | 5 | <0.001 |
|  |  | All Cohort RR = 1 | 128.022519509615 | 16 | <0.001 |
|  |  | All Local Drifts = Net Drift | 107.088964070439 | 12 | <0.001 |
|  | **High-middle SDI** | NetDrift = 0 | 239.533189689152 | 1 | <0.001 |
|  |  | All Period RR = 1 | 394.414027083542 | 5 | <0.001 |
|  |  | All Cohort RR = 1 | 664.988767584443 | 16 | <0.001 |
|  |  | All Local Drifts = Net Drift | 98.3189819855087 | 12 | <0.001 |
|  | **High SDI** | NetDrift = 0 | 7.32148335079382 | 1 | 0.007 |
|  |  | All Period RR = 1 | 62.9456076902872 | 5 | <0.001 |
|  |  | All Cohort RR = 1 | 323.677093053803 | 16 | <0.001 |
|  |  | All Local Drifts = Net Drift | 293.66716574093 | 12 | <0.001 |
| **Male** | **Global** | NetDrift = 0 | 9.75545537540158 | 1 | 0.002 |
|  |  | All Period RR = 1 | 16.6296877410111 | 5 | 0.005 |
|  |  | All Cohort RR = 1 | 97.0126739261652 | 16 | <0.001 |
|  |  | All Local Drifts = Net Drift | 81.3721351699134 | 12 | <0.001 |
|  | **Low SDI** | NetDrift = 0 | 116.062321315819 | 1 | <0.001 |
|  |  | All Period RR = 1 | 118.266192604664 | 5 | <0.001 |
|  |  | All Cohort RR = 1 | 189.982392267118 | 16 | <0.001 |
|  |  | All Local Drifts = Net Drift | 8.23635334875633 | 12 | 0.766 |
|  | **Low-middle SDI** | NetDrift = 0 | 96.9798633207965 | 1 | <0.001 |
|  |  | All Period RR = 1 | 110.289152356298 | 5 | <0.001 |
|  |  | All Cohort RR = 1 | 310.331864350289 | 16 | <0.001 |
|  |  | All Local Drifts = Net Drift | 83.1621850064582 | 12 | <0.001 |
|  | **Middle SDI** | NetDrift = 0 | 42.450429320219 | 1 | <0.001 |
|  |  | All Period RR = 1 | 77.9559107311999 | 5 | <0.001 |
|  |  | All Cohort RR = 1 | 155.277291931261 | 16 | <0.001 |
|  |  | All Local Drifts = Net Drift | 41.9975658941271 | 12 | <0.001 |
|  | **High-middle SDI** | NetDrift = 0 | 76.5583411540137 | 1 | <0.001 |
|  |  | All Period RR = 1 | 101.145747015056 | 5 | <0.001 |
|  |  | All Cohort RR = 1 | 155.736220940676 | 16 | <0.001 |
|  |  | All Local Drifts = Net Drift | 11.4885211752576 | 12 | 0.488 |
|  | **High SDI** | NetDrift = 0 | 1.7749708212948 | 1 | 0.183 |
|  |  | All Period RR = 1 | 24.176291079023 | 5 | <0.001 |
|  |  | All Cohort RR = 1 | 305.393278414215 | 16 | <0.001 |
|  |  | All Local Drifts = Net Drift | 239.520788335297 | 12 | <0.001 |


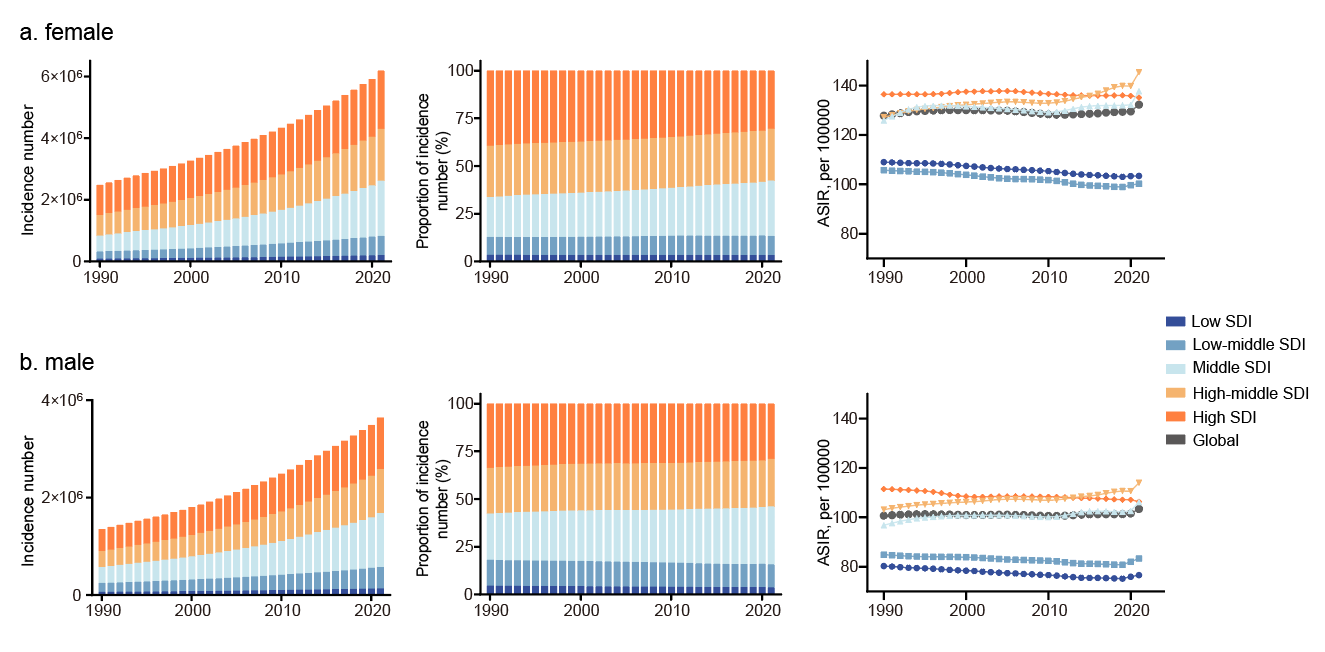


**S1 Fig.** Temporal change in the numbers, proportion and ASIR of ADRD incidence across SDI quintiles for (a) female and (b) male from 1990 to 2021.


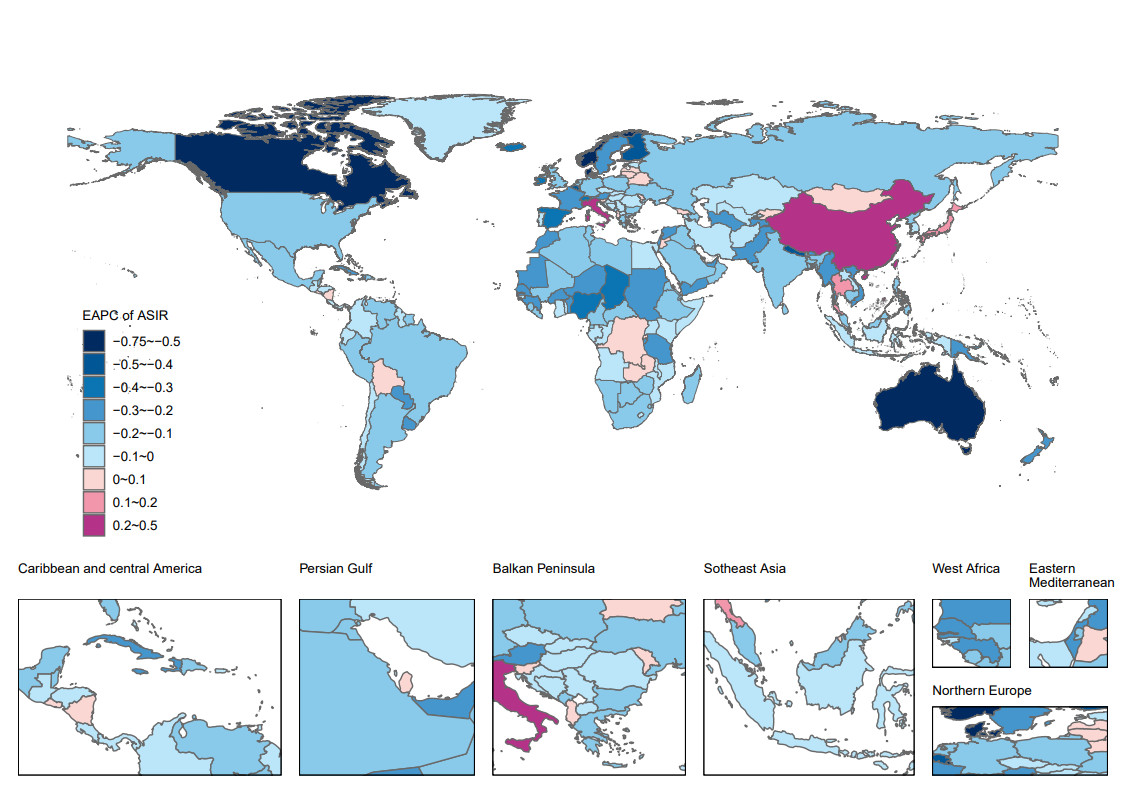


**S2 Fig**. World map of EAPC of ASIR during 1992−2021 for ADRD in 204 countries and territories. Reprinted from *Resource and Environmental Science Data Platform* [<https://www.resdc.cn>], under a CC BY license, with permission from the *Institute of Geographic Sciences and Natural Resources Research, Chinese Academy of Sciences*, original copyright © 2014-2025.


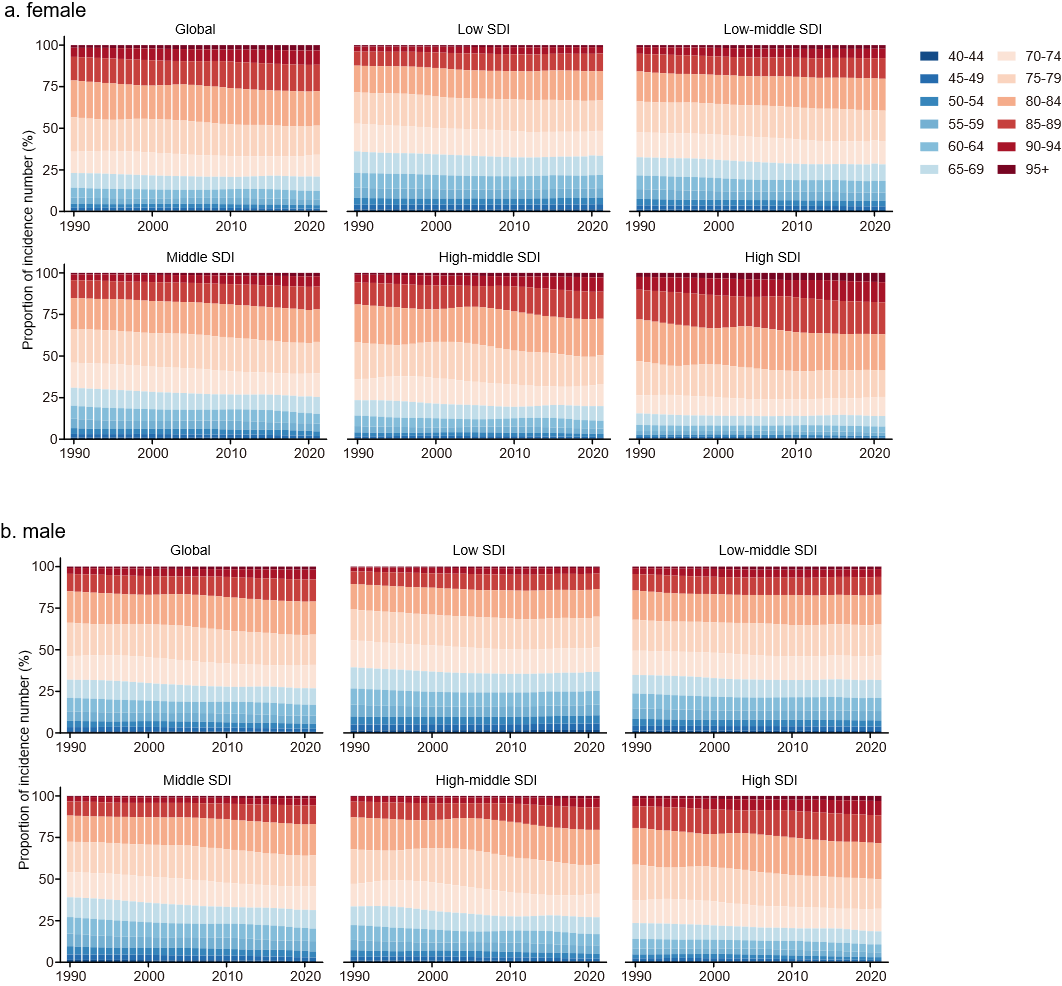


**S3 Fig**. Temporal change in age distribution of ADRD incidence numbers for (a) female and (b) male from 1990 to 2021.


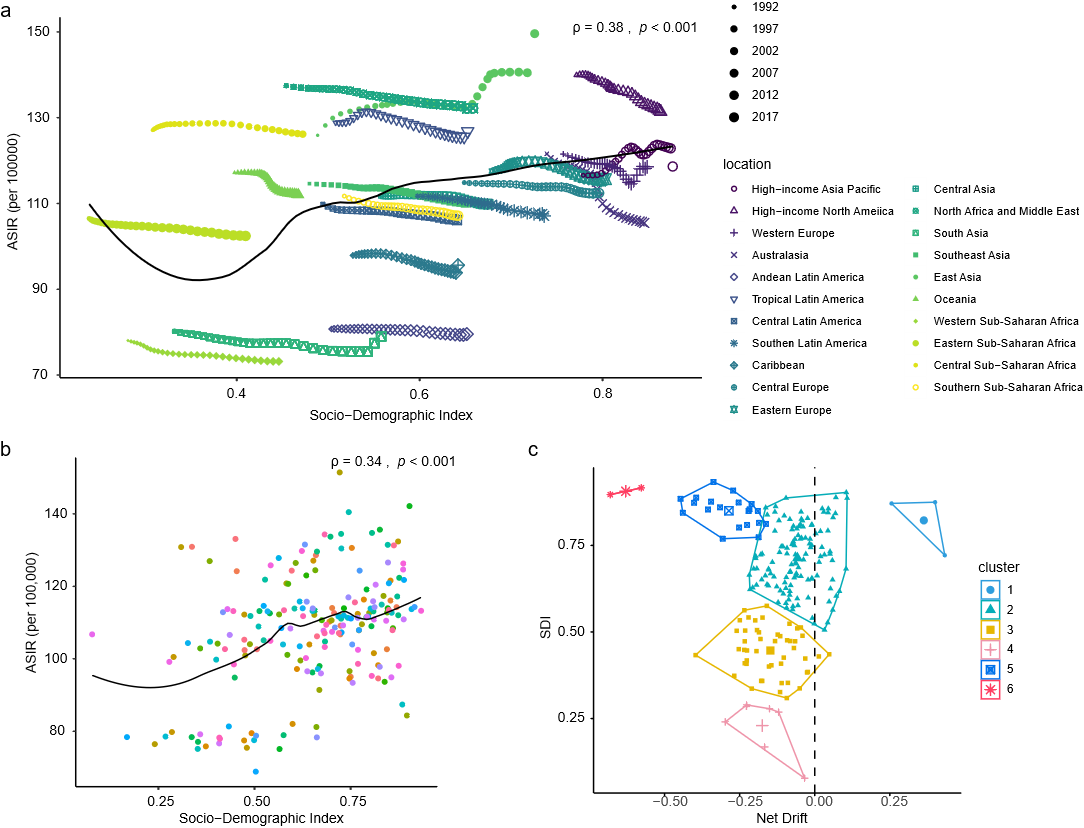


**S4 Fig**. (a) Correlations between the ASIR of ADRD and 21 SDI regions from 1992 to 2021. (b) Correlations between the ASIR of ADRD and 204 countries and territories in 2021. Associations were calculated with Pearson correlation analysis. (c) Cluster analysis of net drift and 204 countries and territories.
